# Public health leadership: a framework inspired by timeless lessons from 500 years of the Jesuit tradition

**DOI:** 10.3389/fpubh.2025.1621381

**Published:** 2025-09-02

**Authors:** Laura Chyu, Erin G. Grinshteyn, Taryn Vian, Timothy Godfrey S.J.

**Affiliations:** ^1^Health Professions Department, School of Nursing and Health Professions, University of San Francisco, San Francisco, CA, United States; ^2^Lone Mountain Global LLP, Auburndale, MA, United States; ^3^Nursing Department, School of Nursing and Health Professions, University of San Francisco, Orange, CA, United States

**Keywords:** Jesuit tradition, public health leadership, health equity (MeSH), global citizenship, spiritual leadership, cross-sectoral partnerships, community engagement, inclusive leadership

## Abstract

The complex and dynamic nature of public health challenges calls for public health leaders who are able to respond with agility, insight, and integrity. In addition to technical expertise and skills, effective public health leadership requires ethical decision-making, collaborative partnerships, and a commitment to justice. Traditional leadership models often fall short in capturing the population-based aspects of public health and integrating a spiritual approach. This conceptual paper examines Jesuit principles and practices and how they can be applied to public health practice and leadership. The proposed framework outlines ten leadership principles: inclusive leadership, service to others, care for the whole person (*cura personalis*), striving for the greater good (*magis*), self-awareness, discernment in decision-making, cross-sectoral and community partnerships, global citizenship, lifelong learning and growth, and adaptability and innovation. This Jesuit-inspired framework offers a spiritual and values-based approach to cultivating compassionate, resilient, and effective public health leaders. It can be applied in academic and workplace settings to strengthen leadership training and guide strategic decision-making. By adopting and adapting Jesuit principles, public health leadership can be conceptualized as an inclusive, mission-driven practice committed to health equity and social justice.

## 1 Introduction

In a time of complex and evolving public health challenges on the global stage, effective and skilled public health leadership is increasingly vital. Within the past 5 years, the world has experienced the COVID-19 pandemic, the reemergence of diseases previously eradicated in certain areas, a worsening climate crisis, and worsening health disparities both in the US and abroad (1–4). Drastic defunding of health research in the U.S. and foreign assistance for public health programs, a steep reduction in personnel at federal health agencies, and reversal of initiatives that address health and social disparities are exacerbating public health concerns (5). Our capacity to address these and future challenges relies on the preparation of a resilient and competent public health workforce, including effective and ethical leaders at every level, to protect and promote population health.

Public health leadership differs from traditional models of leadership in several ways. In contrast to leadership in other settings that center on individual outcomes or organizational performance, public health leadership aims to improve health outcomes for entire populations, focusing on the most marginalized and underserved (6). In addition to leadership goals of efficiency, productivity, and financial sustainability, public health leadership focuses on health equity, social justice, and health as a human right (7–11). Public health leadership actively engages and partners with community members and other stakeholders to build trust and solidarity and to develop solutions (7, 11, 12). Given the complex challenges facing public health now and in the near future, novel approaches and frameworks for leadership are needed in this changing field. This paper will discuss a unique leadership framework that adapts and integrates Jesuit principles and practices for effective public health leadership.

The Society of Jesus, commonly known as the Jesuits, was founded by St. Ignatius of Loyola in 1540 and is one of the world's largest and most influential Catholic orders (13, 14). The origins of the Jesuits can be traced back over 500 years to the Catholic Counter-Reformation, a period of great religious and social upheaval in Europe, and the need to renew and innovate in response. The Jesuits are historically known for their discipline, intellectual rigor, and commitment to social justice (14). Education became one of the Jesuits' most influential tools and “an instrument for justice” (15, 16) with hundreds of schools and universities established worldwide by the early 17th century (13). Today, Jesuit educational institutions are recognized for academic excellence and their mission to develop thoughtful, compassionate, and ethical leaders committed to serving others (14). Public health schools and programs today play an instrumental role in developing and preparing public health leaders and are well aligned with the goals of Jesuit education to prepare skilled and caring leaders.

The Jesuits' global reach and enduring relevance are a testament to a leadership approach grounded in education, community, and service to others. Leadership education is a natural outgrowth of many Jesuit values and practices, particularly leadership that focuses on service and justice. The leadership of Pope Francis, the first Jesuit Pope, provides a compelling contemporary example of leadership rooted in the Jesuit tradition. His papacy was notable for its hallmark traits of the Jesuit tradition, including a commitment to service, a preferential focus on the marginalized and oppressed, inclusivity, and a global perspective, which distinguished him from his predecessors (17). The approach of the Jesuit tradition can be applied to prepare dynamic and resilient public health leaders in the 21st century to promote the health and wellbeing of individuals and communities. The deeply spiritual nature of the Jesuit tradition can serve as a guiding force to cultivate empathy and compassion in public health practice, foster an understanding of the interconnectedness of our global community, and work toward dismantling systemic injustices. While the Jesuit tradition is rooted in the Catholic faith, its values and practices can be adopted by individuals of any or no religious affiliation. Integrating a spiritual component into leadership practice, regardless of religious affiliation or belief, can enhance one's sense of purpose, meaning, and connection, while also strengthening commitment to one's profession or community (18, 19).

## 2 Public health leadership framework

**Figure 1:**
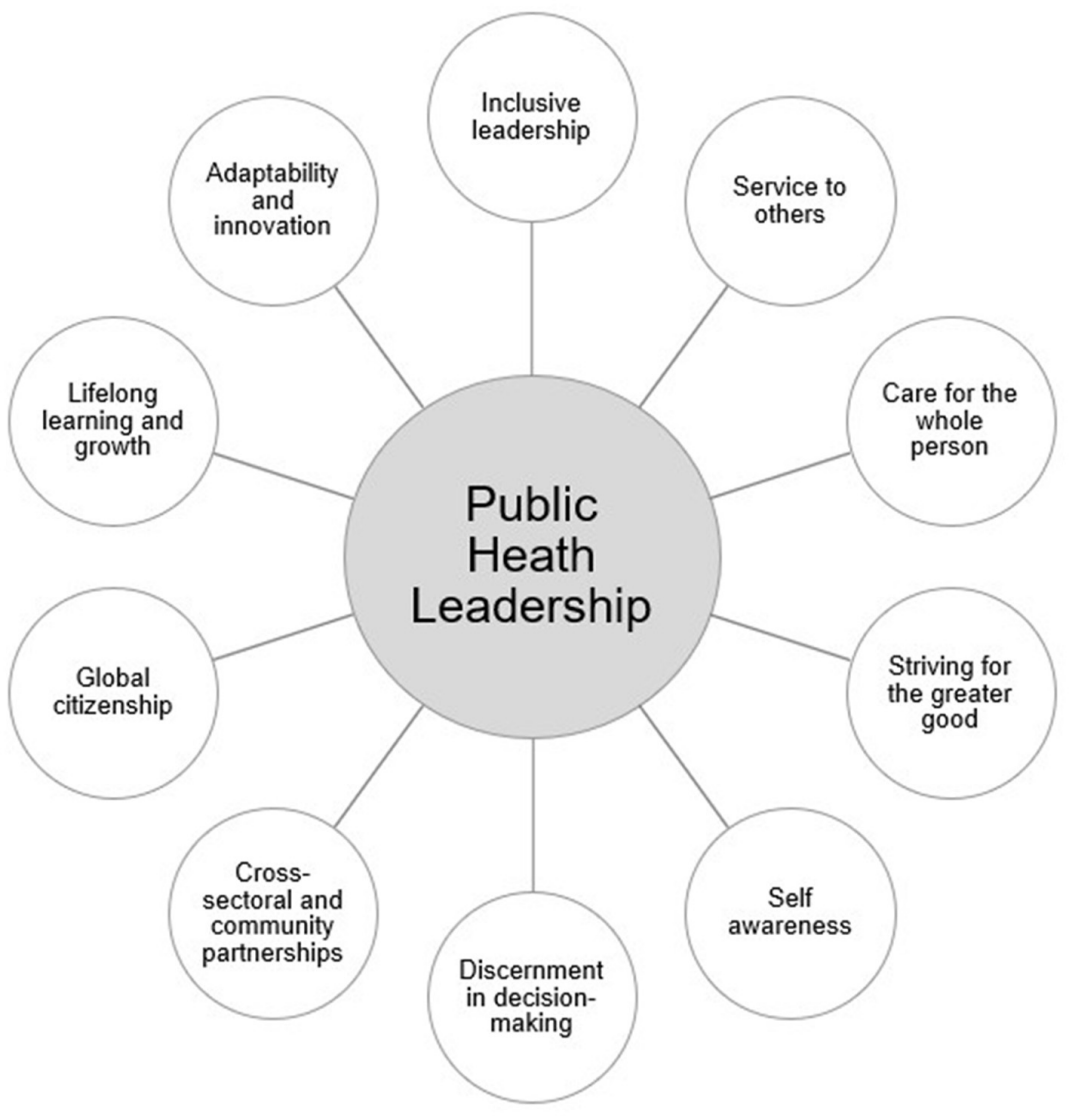
A framework for public health leadership inspired by the Jesuit tradition.

The Jesuit tradition, with its emphasis on service, discernment, inclusivity, and care for the whole person, offers a philosophy that closely aligns with the core values and mission of public health. To identify key Jesuit principles relevant to public health, the authors drew upon both their professional experience within the Jesuit university context and a review of diverse sources, including peer-reviewed journal articles, periodical articles, books, institutional websites, and papal encyclicals. Ten core Jesuit principles were selected and examined for their applicability to public health leadership and practice. Together, these concepts comprise a public health leadership framework that integrates spiritual wisdom from the Jesuit tradition with practical strategies for advancing health and social equity (Figure 1). Table 1 summarizes each Jesuit principle and its application to public health leadership.

**Table 1:** Jesuit principles and their application to public health leadership.

Leadership principle^*^	**Jesuit tradition/Principle**	**Application to public health leadership**
2.1) Inclusive leadership	Every individual has the capacity to lead within their unique roles and contexts	• Recognition and authentic engagement of people at all levels in public health practice; • Promote leadership development among individuals from communities directly impacted by public health challenges
2.2) Service to others	Being a person for and with others, particularly prioritizing the needs of those who are marginalized or oppressed	• A commitment to public service to advance health equity and improve population health, with a focus on addressing the needs of historically marginalized and underserved groups
2.3) Care for the whole person	*Cura personalis*	• Holistic and multidimensional definitions of health for individuals and communities; • Cross-sectoral leadership development addressing health in all policies; • Respect for the dignity, strengths, history, values, and lived experiences of communities
2.4) Striving for the greater good	*Magis*	• Ethical decision-making that prioritizes the public good, especially for historically marginalized and underserved communities
2.5) Self-awareness	Reflection as a spiritual and intellectual practice	• Reflection to understand one's values, strengths, weaknesses, biases, positionality, and implications of one's actions on communities served
2.6) Discernment in decision-making	Spiritual process used to make thoughtful and ethical decisions	• Ongoing practice of reflection and integration of diverse perspectives and sources of information, including community insight, to make well-informed decisions
2.7) Cross-sectoral and community partnerships	Strategic partnerships across sectors; accompaniment with communities	• Collaborative and deliberate efforts to develop multi-sectoral partnerships with government agencies, nonprofit organizations, the private sector, faith-based organizations, and academic institutions to address social and structural determinants of health • Community partnerships that meaningfully engage community members and build community capacity and leadership to strengthen community wellbeing
2.8) Global citizenship	Care for each other and our mutual home	• Global collaboration, solidarity, and shared responsibility for the human health and environmental health of all
2.9) Lifelong learning and growth	Contemplatives in action	• Acquisition of new skills and knowledge • Development of new partnerships across sectors and disciplines • Reflection to inform ethical and compassionate action for the common good
2.10) Adaptability and innovation	Flexibility and ingenuity in a changing world	• Creative and collective problem-solving using innovative skills and strategies to address rapidly evolving public health challenges • Openness to adjusting approaches in response to unique contexts and in collaboration with diverse stakeholders

^*^Each leadership principle is numbered to correspond with the section number in this article.

### 2.1 Inclusive leadership

The Jesuit tradition recognizes that every individual holds the potential to influence and lead others (13). The Jesuits emphasize equipping individuals with the skills and capacity for independent discernment to inform thoughtful action. In this expanded vision of leadership, everyone has the capacity to lead within their unique roles and contexts. Leadership can be dramatic, highly visible, and result in large-scale change; at other times, leadership can be discreet, relational, and measured through more personal or community-level impact. Effective leadership can take different forms and adapts to the needs of the situation in which they arise (20).

This inclusive approach to leadership is highly relevant to public health leadership given the broad scope of public health work and commitment to reducing inequities, which requires constituent and stakeholder involvement. Kouzes and Posner (21) discuss how everyone has the capacity to lead in different ways and contexts, including at work, in communities, in schools, or through personal relationships.. Public health leadership does not require a formal title but can be practiced in every position, role, function, and context if a person acts on available opportunities to make an impact. For example, frontline community health workers exhibit leadership when working with community members to build trust and provide support and services to the community. Those without formal leadership titles within a public health organization, such as advocates, volunteers, and community members, also demonstrate leadership by raising awareness about public health issues and sharing their lived experiences. An inclusive approach to public health leadership actively recognizes and values diverse perspectives, experiences, and identities within teams and communities (22). It fosters environments where all individuals feel respected, heard, and supported in contributing meaningfully in different ways, including conducting research, decision-making, planning, and policymaking (11, 23). By authentically engaging people at all levels and developing leadership capacity within communities, especially those that have been historically marginalized or underrepresented, trust and shared ownership develop (24). An inclusive leadership approach yields more equitable, culturally responsive, sustainable, and innovative programs and policies, and ultimately improved health outcomes (22, 25).

### 2.2 Service to others

A cornerstone of the Jesuit spiritual tradition is serving others as an expression of faith and justice. During his papacy, Pope Francis emphasized the importance of “a faith that does justice,” meaning a faith that compels one to actively work to promote justice and advocate for the marginalized and disadvantaged (15). He tirelessly advocated for and served the poor, migrants, refugees, the incarcerated, indigenous peoples, and other marginalized groups (26). The former Superior General Rev. Pedro Arrupe articulated this fundamental aspect of the Jesuit spiritual tradition as “being persons for and with others,” urging individuals to aspire beyond self-interest and success and to use their strengths, education, and influence for the wellbeing of others (27, 28). In particular, service to others should prioritize the needs of those who are marginalized or oppressed in the pursuit of human dignity and the common good. Service in the Jesuit tradition goes beyond acts of charity and emphasizes practicing humility, solidarity, and accompaniment (29). Being a person for and with others means recognizing our shared humanity, listening with empathy, and challenging systems and structures that perpetuate injustice. This value has long been nurtured as a key characteristic of students graduating from Jesuit schools and universities (14, 30).

The Jesuit ethos of service aligns closely with the mission of public health, which is fundamentally based in public service to promote health equity and improve the health and wellbeing of entire populations (11). Public health seeks to address the root causes of social and health disparities, with attention to social and structural determinants of health and the needs of those who have experienced the greatest disadvantage (31). As stewards of community health and wellbeing, public health leaders lead through solidarity and collaboration within and across communities, sectors, and disciplines to work toward creating conditions where everyone can thrive (7). Historically, there have been long-standing partnerships between public health organizations and faith-based communities, in part due to their shared mission to serve others and, particularly, those most in need (32). Greenleaf also noted that the idea of a leader's primary role as a servant is entrenched in Judeo-Christian writings (33). Service as a key component of public health leadership underscores the importance of understanding community priorities and lived realities and enhancing trust and collaboration.

### 2.3 Care for the whole person

*Cura personalis*, or “care for the whole person”, is a foundational Jesuit value that recognizes the uniqueness and completeness of each individual. This holistic approach to an individual's wellbeing encompasses care for the physical, mental, spiritual, emotional, and social needs of each individual, and recognizes their hopes, aspirations, fears, and struggles (34). The Jesuit tradition of *cura personalis* manifests as “a profound care and responsibility for one another, attentive to each person's circumstances and concerns and gifts” (35). Jesuit leaders and educators are encouraged to engage with others with empathy and respect through authentic connection, intentional support, and appreciation for each individual's lived experiences, values, and potential (14, 30).

Conceptualizing health broadly and holistically at both individual and community levels is foundational to effective public health practice and leadership. The World Health Organization's definition of health as “a state of complete physical, mental, and social wellbeing, and not merely the absence of disease or infirmity” underscores the multidimensional nature of health and its role in enabling people to live meaningful, fulfilling lives (36). Public health research has identified an integrated, holistic model of care that recognizes that physical and mental health are inextricably linked and that our healthcare systems need to be more patient-centered and equitable (37–39). However, a more holistic approach to health needs to be embedded in leadership models if systems are to change. The public health field has long recognized that health is impacted by policies extending beyond traditional public health domains, including education, housing, transportation, and criminal justice (40). Although the “health in all policies” framework and the importance of cross-sectoral collaboration are widely acknowledged, structural changes to enable integrated leadership across institutional silos have been limited, and the success of such collaborations has been mixed (40, 41). To effectively address the broad scope of complex, interrelated health determinants, public health leaders must be trained to think systemically and lead across institutional barriers.

Furthermore, a holistic understanding of communities calls for leadership that values the dignity, strengths, history, values, and lived experiences of those communities, rather than viewing them solely through the lens of health outcomes or risk factors (42, 43). Leadership grounded in cura personalis necessitates a flexible, context-specific approach that embraces the uniqueness of each community and avoids one-size-fits-all solutions. It involves cultural humility, support for initiatives that amplify community assets, and cultivation of leadership within communities (42, 43). This approach not only enhances the effectiveness of public health interventions but also affirms human dignity and equity by striving toward the highest attainable standard of health for all (10).

### 2.4 Striving for the greater good

*Magis* is a Latin term meaning “more” or “greater,” but it does not mean simply doing more work or striving for personal success. In the Jesuit tradition, *magis* is a spiritual and ethical call to pursue what is “conducive to the greater service of God and the universal good,” or “the greater good” through one's decisions and actions (44, 45). Individuals are urged to reflect deeply, discern wisely, and act generously in ways that best serve others and align with Jesuit values. *Magis* emphasizes the quality, depth, and impact of one's work in doing what matters most, rather than simply doing more. While other criteria are factored into decision-making, “the greater good” is a defining outcome of discernment within the Jesuit tradition (44).

Public health is premised on population-level prevention to improve the health and wellbeing of entire populations (46, 47). In public health leadership, *magis* translates into making thoughtful, ethical decisions that prioritize the greater good, conceptualized in public health theory as a public good that benefits communities, especially those that have been historically marginalized and underserved (48). Leaders are encouraged to look beyond short-term fixes and instead invest in sustainable, community-centered solutions that advance health equity and justice (24, 49, 50). With complex public health challenges, it may often not be obvious which choice would serve the greater good. Whether designing a program or policy, allocating resources, or responding to a public health crisis, public health leaders should make decisions based on careful consideration of available data and evidence and consultation with stakeholders. Focusing on the public good as an extension of the greater good is a guiding principle for public health leaders to seek ways to promote health and prevent illness and injury while addressing the social and structural conditions that give rise to health inequities in the first place.

### 2.5 Self-awareness

In the Jesuit context, leadership is built upon a strong internal foundation and is about who a person is as much as what they do (13). Based on the teachings of St. Ignatius, reflection is a foundational spiritual and intellectual practice in which individuals are invited to pause, look inward, and consider their experiences, thoughts, and actions to better understand themselves, find God in all things, and imbue meaning in the human experience. Reflection is a tool for cultivating self-awareness, personal growth, discernment, and connection to one's faith (51, 52). The consistent practice of reflection deepens empathy and prepares individuals to act with integrity in service of the greater good (53).

Some leadership models, including those proposed by Goleman (54), Drucker (55), and George et al. (56), assert that leadership begins with self-awareness and managing oneself. Brown (57) and George et al. (56) further underscore the importance of identifying one's core values and consistently using them to guide decision-making and action. In the context of public health leadership, understanding one's values, strengths, and weaknesses can be cultivated through reflection inspired by the Jesuit tradition. Working with diverse communities requires acknowledging personal biases and positions of privilege or disadvantage and understanding the consequences of one's actions and interactions with others (11, 58). Reflection is a tool that helps public health leaders develop a deeper appreciation and mindfulness of individuals and communities. In addition to developing professional skills and credentials for public health practice, public health leaders should prioritize cultivating personal awareness and management (59, 60). Plante et al. found that adopting a daily reflection process had a significant impact on measures of satisfaction with life and individuals' perceived ability to strive for goals, which are important leadership characteristics (61). Through reflection, public health leaders embark on a lifelong journey of introspection and growth via continuous inquiry into what they have learned and how they can evolve in ways to meaningfully serve others to advance public health goals.

### 2.6 Discernment in decision-making

Discernment is the spiritual process used to make thoughtful and ethical decisions that are informed by reflection (62). In 1548, St. Ignatius developed the Spiritual Exercises, a compilation of prayers, meditations, and contemplative exercises, as a way for people to develop their attentiveness, openness, and responsiveness to God in their lives (62–65). Discernment is a critical part of the process of not just learning but also in translating learning into action (65). Discernment involves paying close attention to internal movements (e.g., inner thoughts, emotions, desires) as well as external movements (e.g., conversations with others, previous experiences, observations of social injustices) to seek clarity and identify the best decision moving forward (63). Rather than relying solely on either impulse or logic, Jesuit discernment is an ongoing process that emphasizes self-awareness, openness to multiple perspectives, and a commitment to what serves the greater good.

Discernment is a critical skill in public health that enables leaders to navigate complex challenges and make ethical, equity-centered decisions. Public health leaders often face situations with no clear answers, such as how to allocate limited resources, prioritize competing needs, or respond to complex and evolving crises (66). Effective public health leaders must integrate diverse perspectives and sources of information, balancing expressed needs and interests of communities with insights derived from data and professional expertise to make the best decision (11, 65). The Jesuit approach to discernment, which can be developed through practices such as reflection and self-awareness, encourages leaders to consider the broader implications of their choices, including ethical, social, psychological, economic, and cultural impacts, as well as potential tradeoffs. Discernment also calls for listening to the voices of affected communities with humility and remaining grounded in the core values of public health. This decision-making process helps ensure that policies and practices are evidence-based, ethically sound, and responsive to the realities of those most affected.

A compelling example is the response of Dr. Mona Hanna-Attisha, a pediatrician and public health advocate, to the Flint water crisis in 2015. After colleagues alerted her to the lack of proper drinking water treatment, she analyzed data from electronic medical records and found a significant increase in children's blood lead levels following the city's change in water source (67). These findings, coupled with personal accounts of Flint residents impacted by the water contamination, informed her understanding of the situation. Driven by her ethical commitment to her patients and the community's wellbeing, and because of the urgency of the situation, she decided to publicly release her non-peer-reviewed findings in a press conference, despite the risk of jeopardizing her professional reputation. Her courageous decision was instrumental in exposing the lead contamination crisis and catalyzing national attention to the issue. Dr. Hanna-Attisha continues to lead long-term recovery efforts and advocate for environmental justice (67, 68).

### 2.7 Cross-sectoral and community partnerships

Throughout history and currently, the Jesuits have strategically partnered across multiple sectors, including education, science, politics, and religious and secular institutions (13, 69). The establishment of an extensive network of schools and universities around the world was often conducted in collaboration with local governments and monarchies, who recognized the Jesuits' role in training civic and religious leaders (69). The Jesuits immersed themselves in the communities they served, learning local customs and traditions and engaging in local political and religious systems (69, 70). This adaptive, community-centered approach built mutual trust and understanding, which in turn allowed the Jesuits to tailor their ministries, educational initiatives, and social programs to the needs of local communities. Political, scholarly, and religious alliances also yielded fruitful collaborations in scientific discourse and social change (70, 71).

Building cross-sectoral and interdisciplinary partnerships is an essential competency of public health leadership to address the wide range of factors that influence population health (72–75). Structural determinants of health, including economic and social policies, power dynamics, governance, and social norms, shape access to and quality of health care, education, employment, housing, transportation, social services, access to healthy food, and the physical and social environment (76, 77). Collaborative efforts with government agencies, nonprofit organizations, the private sector, faith-based organizations, and academic institutions are critical for pooling of resources and expertise and help foster innovative approaches to address the root causes of inequities (72, 74). For example, public health departments have partnered with local governments, economic development agencies, and grocery retailers to develop Healthy Food Financing Initiatives (HFFIs) that address food deserts, food insecurity, and nutrition-related disease (78). These collaborative efforts invest in grocery stores and fresh food outlets, which in turn improve community access to nutritious food and stimulate local economies.

The Jesuits were also deeply engaged with communities through the practice of accompaniment, which is described as to “live and walk beside those whom we serve” (79). Accompaniment is an expression of solidarity and commitment to be present with others, especially those who have been historically marginalized or made vulnerable by structural inequities, as companions on a shared journey (80). Listening without judgment, practicing humility, and being willing to learn are required in accompaniment to foster an authentic relationship, where mutual transformation is possible for both parties (81). Pope Francis prioritized the practice of accompaniment as a way for the Church to be more inclusive and demonstrate solidarity with marginalized individuals and communities (29).

Equally important in public health is active engagement and partnerships with communities, built upon relationships and trust, to improve community health and wellbeing. Community members offer key insight into the unique strengths, needs, and priorities of the community to inform relevant and culturally appropriate programs (75). Approaches that equalize power in agenda-setting and decision-making and build community capacity and leadership foster mutual trust and accountability and lead to more effective and sustained outcomes (11, 24, 42, 43). Aligned with the principles of accompaniment, the field of public health has shifted from an approach of “doing for” toward one of “doing with” (49). By forging sustainable partnerships across sectors and with communities, public health leaders can drive systemic change and advance health and social equity.

It is also important to acknowledge that the Jesuits, as part of the broader European colonial enterprise, were involved in practices that caused significant harm to individuals and communities, including participation in slavery, the operation of Indigenous boarding schools, and instances of clergy sexual abuse (82–85). Engaging meaningfully with communities requires a transparent acknowledgment of these historical injustices and a commitment to listening to and supporting those who have been harmed (83, 84). Such acknowledgment is a critical foundation for processes of healing and reconciliation.

Public health leaders must also acknowledge and address past harms to individuals and communities affected by unethical public health practices. A well-documented example is the Tuskegee Syphilis Study, conducted by the U.S. Public Health Service from 1932–1972, in which Black men in Tuskegee, Alabama, were observed for untreated syphilis without their informed consent (86, 87). Even after penicillin became widely available as treatment, participants were denied treatment. In response to the profound harm caused by the study, several measures have been implemented, including the establishment of health benefits and scholarship programs for surviving participants and their families, and major reform in the ethical oversight of human subjects research ethical standards (86, 88, 89). Nevertheless, the enduring legacy of the study, particularly its contribution to the deep mistrust of public health and medical institutions among African American communities, underscores the need for continued community engagement, transparency, and trust-building efforts (87, 90, 91).

### 2.8 Global citizenship

Global citizenship is rooted in the Jesuit spiritual tradition philosophy that we are all part of a shared human family and are called to act with compassion and responsibility for our planet and all its people (92). It also requires individuals to deepen their awareness of their place and responsibility in the world, and understand that our choices and actions have global implications. Global citizenship reflects a deep commitment to solidarity with people around the world, especially those facing injustice and oppression (93). Pope Francis‘ encyclical *Laudato si'* presented the concept of an integral ecology, in which there is a moral imperative for the world's citizens to care for our shared home and for each other (92). This worldview is centered on human dignity and the pursuit of a more humane world and sustainable planet.

This philosophy aligns closely with public health practice, which is inherently embedded in complex global systems and ecosystems. The COVID-19 pandemic illustrated the global nature of health determinants and outcomes as well as the need for better understanding and implementation of a system that prioritizes global citizenship (94). Public health leaders who embrace global citizenship understand that we live in an increasingly interconnected world in which disease, environmental threats, and social inequities do not respect borders. In the public health field, there has been a meaningful shift from terms like “international health,” which can be perceived as something happening across borders, to “global health,” which can be conceptualized more as a collective and unifying phenomenon (95). The emerging field of “planetary health” emphasizes the interdependent and inseparable connection between human health and the health of our global environment (96). Meaningful solutions to public health challenges require global collaboration, cultural humility, and shared responsibility (11). As global citizens, public health leaders act in solidarity with communities around the world to build a more just, sustainable, and healthy future for all.

### 2.9 Lifelong learning and growth

Lifelong learning and growth are deeply embedded in the Jesuit tradition and nurture intellectual curiosity, joy of learning, and deepening one's understanding of self, others, and the world. Jesuit education goes beyond acquiring knowledge and aspires to “learning how to learn, to desire to go on learning all through life” (51, 97). Jesuit institutions cultivate this mindset by developing contemplatives in action, a practice that St. Ignatius described as an interchange between reflection and action to engage with the world more meaningfully (51, 98). Being a contemplative in action means taking time to pause and reflect on what is happening within oneself and in the world, and then responding with thoughtful and compassionate action. It helps individuals stay rooted in their beliefs and values while evaluating and adapting to evolving circumstances (98).

A commitment to lifelong learning and growth positions public health leaders well to tackle complex issues and implement innovative solutions. Continuous learning can entail reimagining health systems; acquiring new knowledge and skills; using novel data sources, analytical methods, and tools; and forging new partnerships across sectors and disciplines. In the face of persistent complex public health challenges and amid ongoing sociopolitical instability, the need for agile, forward-thinking public health leadership has never been more critical. As health determinants, systems, and outcomes rapidly evolve, so too must our leaders by developing new skills and strategies to meet emerging challenges and protect the wellbeing of communities (73, 74). Public health leaders are required to make effective decisions often under pressure; being contemplatives in action balances reflection with informed and decisive action carried out with intention, integrity, and compassion.

### 2.10 Adaptability and innovation in a constantly changing world

Since their inception, the Jesuits have demonstrated remarkable adaptability and innovation by not limiting themselves to a single type of ministry or location. Lowney identified “ingenuity” as a core pillar of Jesuit leadership, describing how the Jesuits confidently adapted to a changing world by exploring new ideas, approaches, and cultures (13). They quickly expanded their work across continents and became cultural bridges, learning local languages and cultures to more effectively share knowledge and offer spiritual guidance (13, 69). Their openness to adapting their methods based on context allowed them to build deep relationships and exert lasting influence in many regions of the world. As pioneers in education and science, the Jesuits created a global network of schools and universities and curriculum that incorporated scientific discovery and the arts (30). Their commitment to forward-thinking education made the Jesuits innovators in both religious and secular spheres.

The Jesuit legacy of adaptability and innovation serves as inspiration for public health leaders. In a field characterized by expansive and complex challenges, public health leaders must exercise adaptive leadership strategies, including quickly grasping the type and complexity of problems, soliciting diverse perspectives, promoting shared responsibility and collective problem-solving, and confidently guiding others through change (99, 100). This requires the ability to skillfully navigate unfamiliar and uncertain terrain and actively seek opportunities to adapt, innovate, and drive progress. Like the Jesuits, public health leaders must be willing to listen, learn, and adjust their strategies in response to unique and evolving circumstances and in collaboration with diverse stakeholders (20). By remaining anchored by their values yet flexible, public health leaders can create innovative and sustainable solutions to address the dynamic nature of public health challenges.

An example of adaptability and innovation in public health is the expanded adoption of harm reduction strategies to address the opioid crisis, a deadly epidemic which has resulted in 806,000 opioid overdose deaths in the U.S. since 1999 (101). As it became evident that abstinence-only and punitive approaches were insufficient in curbing opioid overdose deaths, public health leaders have increasingly turned to harm reduction strategies as a more compassionate approach that meets users where they are and provides needed services (102–104). Harm reduction strategies include the distribution of naloxone (a medication that can rapidly reverse the effects of an opioid overdose), implementation of safe syringe programs, drug checking services (allowing people to test their illicit drugs for unknown and potentially harmful substances, such as fentanyl, before use), and supervised consumption sites (103, 105–109). Together with abstinence approaches, these strategies make up an integrated continuum of care. While some harm reduction strategies have existed for decades and others are more recent innovations, they collectively reflect public health's capacity to adapt to a complex and rapidly evolving crisis. This shift has required public health professionals to challenge stigma, collaborate across sectors (e.g., health care, law enforcement, housing), and prioritize person-centered care that upholds the dignity and autonomy of the individual (102, 106, 107).

## 3 Discussion

This paper presents a leadership framework inspired by the Jesuits and their religious, philosophical, and spiritual tradition that has endured for nearly five centuries. The Jesuit tradition offers a compelling model for mission-driven leadership in public health. In public health, aligning actions to public health's core mission—advancing health equity, social justice, and the common good—provides a clear and unified direction for all public health professionals. This approach ensures that decisions are anchored in shared values and consistently focused on long-term impact, and not on just what is expedient. The ten Jesuit principles and practices that comprise the framework remain highly relevant today in public health leadership. Public health challenges are uniquely complex and high-stakes, often unfolding on a large scale, involving multiple stakeholders, and under public scrutiny in the media. Solutions must address immediate crises as well as shape long-term health consequences for communities and future generations (110). The framework serves as a practical and meaningful guide for public health leaders to navigate complex and dynamic public health challenges. It moves beyond conventional leadership models that focus on skills and status and reframes leadership as an active commitment to justice, collaboration, and service for the public good, deepened by spiritual practice. A mission-driven approach to leadership in public health is particularly salient in times of crisis or uncertainty by helping leaders stay grounded and make thoughtful and ethical decisions rather than reactive ones. Ultimately, these principles can strengthen public health efforts at local, national, and global levels and build a competent, resilient, and compassionate public health workforce.

Application of this framework for leadership development can be implemented in educational and workplace settings and organizational culture to support a continuous process of learning and growth (53). In public health schools and programs, leadership curriculum should expand beyond skills and competencies and underscore mission-driven leadership approaches that reflect the unique context and values of public health. For example, integrating reflective practices, such as self-awareness exercises and discernment, into coursework equips students with tools they can carry into their careers and personal lives (19, 59, 111). Similarly, workplaces can play an important role by embedding leadership development into their organizational cultures. This includes authentically engaging employees and communities as collaborators in the design, implementation, and evaluation of public health initiatives (24, 112). Workplaces can also reinforce lifelong learning and growth by offering workshops on new skills and topics, certification opportunities, and support for continued education. Both schools and workplaces can model care for the whole person by providing resources to support physical, mental, social, and spiritual wellbeing (81, 113–115).

Future efforts should focus on operationalizing this framework and evaluating its effectiveness in different settings. This includes developing tools to measure various dimensions of public health leadership and identifying best practices for implementation and adaptation. Rooted in the Jesuit tradition of reflection, justice, and service, this leadership framework offers timely insights for shaping the next generation of public health leaders.

## Data Availability

The original contributions presented in the study are included in the article/supplementary material, further inquiries can be directed to the corresponding author.
